# Nutritional Management for Crohn’s Disease

**DOI:** 10.3390/nu16162597

**Published:** 2024-08-07

**Authors:** Sara Sila, Iva Hojsak

**Affiliations:** 1 Referral Center for Pediatric Gastroenterology and Nutrition, Children’s Hospital Zagreb, 10000 Zagreb, Croatia; sara.sila0810@gmail.com; 2 Department of Pediatrics, School of Medicine, University of Zagreb, 10000 Zagreb, Croatia; 3 Department of Pediatrics, Faculty of Medicine Osijek, Josip Juraj Strossmayer University of Osijek, 31000 Osijek, Croatia

Diet has been implicated in the pathogenesis of inflammatory bowel disease (IBD) and, more specifically, Crohn’s disease (CD), for a long time. Diet in connection to IBD first sparked interest after an observation that immigrants who moved from low- to high-income countries with more westernized diets increased their risk for the development of the disease [1]. Afterward, with the discovery of exclusive enteral nutrition (EEN) as a treatment for CD, diet gained attention not only as a risk factor for the development of IBD but also for its management. Although research undoubtedly points to diet being one of the major modifiers for disease development and/or treatment, this relationship is very complex and not yet fully elucidated [2].

Research studies presently aim to address two main objectives concerning diet and CD: (1) to identify dietary habits and/or food constituents that are associated with a higher risk of acquiring CD in susceptible individuals (pre-illness diet) and (2) to identify dietary habits and components of a diet that can affect the course of the disease and potential dietary treatment of CD.

We know from epidemiological studies that diet plays an important role in the development of IBD. More specifically, a diet high in polyunsaturated fatty acids (PUFAs), n-6 fatty acids, and meat is associated with an increased risk of developing IBD. By contrast, a high intake of dietary fiber and fruits is associated with a lower risk of developing CD [3]. Moreover, recent studies have suggested that processed food, especially ultra-processed food, might also be a risk factor for the development of IBD [4,5]. Importantly, the role of diet in the prevention of the disease has been recognized in the recent guidelines of the European Society for Clinical Nutrition and Metabolism (ESPEN), which recommends a diet rich in fruit, vegetables, and n-3 fatty acids, as well as low in n-6 fatty acids and ultra-processed foods [6].

Significant progress in the nutritional management of CD has been made in previous decades, especially since the discovery of exclusive enteral nutrition (EEN), which is suggested as a first-line treatment in pediatric patients with mild active CD today [6,7]. Although EEN leads to remission in up to 90% of patients with luminal CD, it has its limitations—it is not routinely used in adult patients, and, since the patient should consume liquid formulas as their sole source of nutrition for a minimum of 6 weeks, it is also very demanding. Therefore, novel nutritional interventions have been thoroughly investigated in recent years. These are mostly based on reduced exposure to dietary components that could adversely affect the microbiome and intestinal barrier. The most promising so far is the Crohn’s disease exclusion diet (CDED), a whole-food diet coupled with partial enteral nutrition (PEN), which has been shown to have similar efficacy outcomes to EEN but is better tolerated by the patient [8]. Moreover, the diet induced sustained remission in a significantly higher proportion of patients than EEN at week 12 [8]. Its efficacy has been proven in both children and adults and has been recommended in the ESPEN’s recent guidelines as a possible alternative to EEN for treating mild to moderate CD [6].

Although research clearly points to the fact that diet plays an important role in the pathogenesis of CD, the relationship is not yet fully elucidated. The current dogma of the pathogenesis of CD recognizes the interaction between environmental factors (diet) and the gut microbiota in individuals with a genetic predisposition. Evidence now proposes that genetics can only explain a small fraction of the risk of IBD development, with a rising evidence base linking the diet to disease occurrence [9,10]. Moreover, a proportion of patients will not respond to a contemporary drug or dietary therapy [11]. Consequently, attention has recently shifted to stratified or precision medicine, where one uses patient information to decide the optimal treatment for this group (stratification) or individual patient (precision) [12]. Therefore, “one size fits all” most probably does not apply only to the development, but also to the treatment of CD since multiple factors and mechanisms are likely in play.

Many questions remain unanswered. Future research should focus on different aspects of the diet that are associated with CD. First, causality between diet, microbiome, and IBD must be established, as well as the mechanism of action of currently available dietary treatments such as EEN and CDED. Understanding the disease’s mechanism of development and/or treatment is a prerequisite for successful prevention and/or management. Next, efforts should be made to conduct future studies that will help stratify patients by disease phenotype, gut microbiota characteristics, and dietary intake to predict responders and non-responders to different treatment options. Moreover, despite the fact that available data support dietary treatments for remission induction in patients with active CD, few data exist regarding optimal diets, which will help sustain the remission of the disease. Similarly, limited data exist regarding the combination of standard medical and dietary treatment, such as biologics and the CD exclusion diet or PEN. Moreover, future studies should also focus on the role of diet in treating ulcerative colitis.

Taken together, it is evident that diet research in IBD is both growing and quickly evolving. Therefore, focus should be placed on high-quality research, especially on clinical studies that will confirm the efficacy of current, but also developing, novel dietary treatment options.
